# Neuropathological Changes in Dementia With Lewy Bodies and the Cingulate Island Sign

**DOI:** 10.1093/jnen/nlz047

**Published:** 2019-05-24

**Authors:** Lina Patterson, Michael J Firbank, Sean J Colloby, Johannes Attems, Alan J Thomas, Christopher M Morris

**Affiliations:** 1 Alzheimer’s Society Doctoral Training Centre, Newcastle University, Newcastle upon Tyne, UK; 2 Institute of Neuroscience, Newcastle University, Newcastle upon Tyne, UK; 3 Newcastle upon Tyne Hospitals NHS Foundation Trust, Newcastle upon Tyne, UK; 4 Gateshead Health NHS Foundation Trust, Queen Elizabeth Hospital, Gateshead, Tyne and Wear, UK; 5 NIHR Biomedical Research Centre Newcastle, Newcastle University, Newcastle upon Tyne, UK

**Keywords:** α-Synuclein, Alzheimer disease, Cingulate island sign, Dementia with Lewy bodies, Single-photon emission tomography (SPECT)

## Abstract

The cingulate island sign (CIS) refers to the relative sparing of metabolism in the posterior cingulate cortex (PCC) and represents an important biomarker in distinguishing dementia with Lewy bodies (DLB) from Alzheimer disease (AD). The underlying basis of the CIS is unknown; therefore, our aim was to investigate which neurodegenerative changes underpin the formation of CIS. Using quantitative neuropathology, α-synuclein, phosphorylated Tau, and amyloid-β pathology was assessed in 12 DLB, 9 AD and 6 age-matched control patients in the anterior cingulate (ACC), midcingulate, PCC, precuneus/cuneus and parahippocampal gyrus. All participants had undergone ^99m^Tc-hexamethylpropyleneamine oxime (HMPAO) single-photon emission computed tomography imaging during life to define the presence or absence of CIS. In the DLB group, no significant correlations were observed between CIS ratios and neurodegenerative pathology in PCC. In DLB, however, the ACC showed lower HMPAO uptake, as well as significantly higher α-synuclein and amyloid-β burden compared with PCC, possibly underlying the relative preservation of perfusion in PCC when compared with ACC. Our findings suggest that neurodegenerative pathology does not directly correlate with the CIS in DLB, and other metabolic or pathological changes are therefore more likely to be relevant for the development of the CIS.

## INTRODUCTION

Dementia with Lewy bodies (DLB) is second only to Alzheimer disease (AD) in terms of prevalence, accounting for ∼15%–20% of all neuropathologically defined dementia patients (1). Clinically DLB is characterized by progressive dementia, parkinsonism, fluctuating cognition, recurrent complex visual hallucinations, and rapid eye movement sleep behavior disorder (2). Early and accurate diagnosis of DLB is essential for optimal management of the disorder (2–4). Several biomarkers are used clinically in the differential diagnosis of DLB, with dopamine transporter imaging proven to be effective in detecting early nigrostriatal changes (5, 6) and [^123^I]metaiodobenzylguanidine myocardial scintigraphy to detect changes in the peripheral sympathetic nervous system (7). The cingulate island sign (CIS) is a supportive biomarker in discriminating DLB from AD according to diagnostic criteria (2, 8, 9), and refers to the relative preservation of posterior cingulate cortex (PCC) metabolism, in relation to precuneus and cuneus (Pr/Cu) metabolism, and is commonly observed in DLB patients (10, 11). This can be identified using ^18^F-fluorodeoxyglucose (FDG) positron-emission tomography (PET) imaging and also using hexamethylpropyleneamine oxime (HMPAO) single-photon emission computed tomography (SPECT) as a marker of perfusion to define the CIS (12).

A pathological hallmark of DLB is aggregation of α-synuclein protein, the major component of Lewy bodies and Lewy neurites found in widespread cortical and subcortical brain regions (13). Neuropathological characteristics of AD are defined by the presence of extracellular aggregation of amyloid-beta (Aβ) protein in the form of senile plaques and perivascular amyloid, as well as neurofibrillary tangles (NFT) and neuropil threads, composed of hyperphosphorylated microtubule associated protein Tau (p-Tau). DLB and AD patients share many clinical and pathological characteristics, with some coexisting AD pathology observed in 50%–80% of pathologically confirmed DLB patients (14). Clinico-pathological correlation has been able to demonstrate that biomarkers of DLB, such as reduced dopamine transporter uptake in the striatum is due to pathological change in the midbrain substantia nigra (15). Similarly, reduced cardiac [^123^I]metaiodobenzylguanidine uptake in DLB may have as its basis decreased cardiac sympathetic innervation due to degeneration of noradrenergic nerve fibers (16). How pathology affects the presence of a specific biomarker such as CIS is therefore a key question in determining the validity of a biomarker.

The semiquantitative scoring of pathology using NFT Braak staging has been suggested to be a correlate of the CIS using FDG-PET (10), with CIS in DLB thought to be reflective of lower p-Tau pathological burden, although the intrinsic pathological basis of the CIS is unknown. While FDG-PET imaging has been shown to be more sensitive in detecting CIS (12), SPECT imaging is more widely used for clinical screening and examination of patients with dementia (17), with high sensitivity in detecting differences in cerebral perfusion between AD and DLB (8, 17). We therefore determined how different cingulate pathologies relate to CIS assessed using Technetium-99m (^99m^Tc)-HMPAO SPECT imaging in a cohort of clinically and pathologically characterized group of older individuals.

## MATERIALS AND METHODS

### Cohort

Patients and control donors were part of longitudinal studies undertaken at Newcastle University. Ethical approval for the study was granted by the Newcastle and North Tyneside National Health Service (NHS) Research Ethics Committee. All patients had received physical, neurological and neuropsychiatric examinations during life. All patients had consented to the use of their brain tissue for research purposes and next of kin gave consent to donate after death. Postmortem neuropathological assessment was performed according to standardized neuropathological diagnostic procedures, which together with clinical data was used to make a clinicopathological diagnosis (2). Brain tissue was obtained from the Newcastle Brain Tissue Resource (NBTR), a UK Human Tissue Authority-regulated research tissue bank.

**FIGURE 1.**
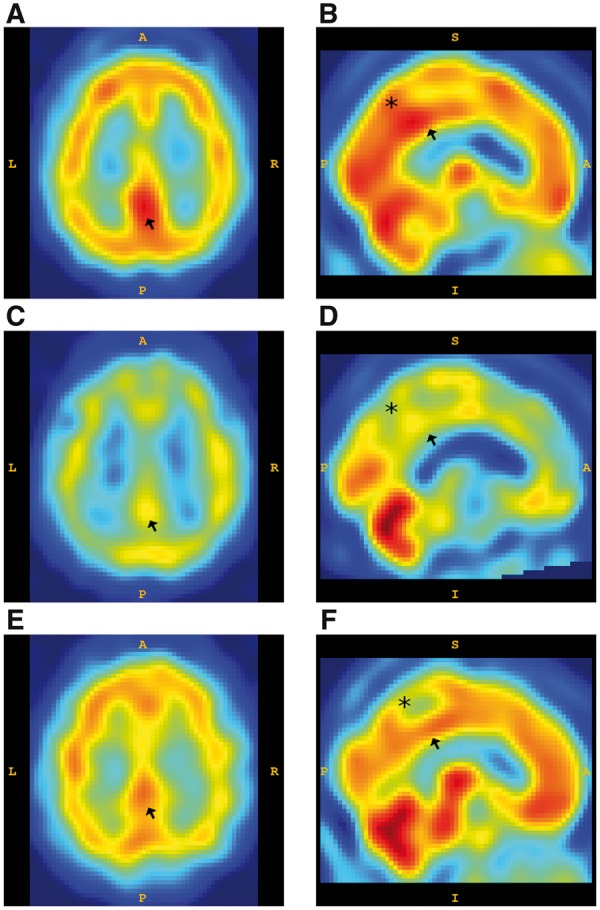
^99m^Tc-HMPAO SPECT perfusion images from control, Alzheimer disease, and dementia with Lewy bodies donors. Representative axial slice images **(A, C, E)** through the posterior cingulate and **(B, D, F)** midline sagittal slice images of perfusion determined using ^99m^Tc-HMPAO SPECT. In older normal patients **(A, B)** without dementia, the posterior cingulate cortex (arrow) and precuneus (asterisk) appear relatively bright. In AD patients **(C, D)**, perfusion intensity is reduced in both these structures. In DLB patients **(E, F)**, the precuneus shows a greater reduction compared with the posterior cingulate cortex, which shows relatively preserved perfusion. (L, left; R, right; A, anterior; P, posterior; S, superior; I, inferior.)

Patients were diagnosed in life as having DLB (n = 12), AD (n = 9) or were neurologically and psychiatrically normal (n = 6). Standardized neuropathological assessment was according to internationally accepted criteria (1, 18, 19). Of note, 4 DLB patients also fulfilled the neuropathological criteria for high AD neuropathological change and could therefore be classified as neuropathologically mixed AD/DLB with a Lewy body disease (LBD) clinical phenotype (20, 21). All patients had undergone ^99m^Tc-hexamethylpropyleneamine oxime (^99m^Tc-HMPAO) SPECT scans during life. ^99m^Tc-HMPAO is a radioactive tracer which is injected intravenously, taken up in the brain in proportion to cerebral perfusion, and the pattern of its distribution is determined with SPECT imaging with a spatial resolution of ∼10 mm (Fig. 1). Ten individuals with DLB (3 of which were mixed AD/DLB patients), 8 AD and 6 normal controls also had magnetic resonance imaging (MRI) scans, with cortical thickness and volumes taken from the nearest MRI to death. ^99m^Tc-HMPAO SPECT and MRI scans were obtained from previously published studies (22–24). The Mini-Mental State Examination (MMSE), Unified Parkinson's Disease Rating Scale (UPDRS) scores and duration of illness were recorded at the time of SPECT. Cerebral perfusion and MRI volume data were acquired for the anterior cingulate cortex (ACC), midcingulate cortex (MCC), PCC, precuneus/cuneus (Pr/Cu), and parahippocampal gyrus (PHG).

### Postmortem Tissue Samples

At autopsy, the right hemisphere was fixed in 10% formalin for 4–6 weeks. Following fixation, the hemisphere was cut into 7-mm coronal slices then dissected into blocks and embedded in paraffin wax for neuropathological assessment. Paraffin embedded tissue blocks selected for analysis corresponded to ACC (Brodmann area: BA 24, at the level of the genu of the corpus callosum), MCC (BA 24′, coronal level of the primary motor cortex), PCC (BA 23, coronal level of the splenium of the corpus callosum), precuneus (BA 40, coronal level of supramarginal gyrus), and PHG (BA 28, coronal level of lateral geniculate nucleus) (25). For α-synuclein the ethylenediamine tetra-acetic acid (EDTA) heat-induced antigen retrieval pH 8.0 was performed in a pressure cooker for 2 minutes, followed by 15 minutes in absolute formic acid at room temperature. The citrate heat-induced antigen retrieval pH 6.0 for 10 minutes at full power in an 800 W microwave was carried out for p-Tau, whereas 1 hour formic acid antigen retrieval for Aβ. Ten-micrometer sections (1 per region of interest) were immunostained with primary antibodies to α-synuclein (KM51 anti-α-synuclein, Leica Biosystems, Buffalo Grove, IL, 1:250), p-Tau (AT8 antiphosphorylated tau, Autogen, Holliston, MA, 1:4000), and Aβ (4G8 anti-amyloid-β peptide, Covance, Princeton, NJ, 1:15 000). Immunolabeling was detected using the Menarini X-Cell-Plus HRP Detection Kit (Menarini, Wokingham, UK) and visualized using diaminobenzidine (Menarini) substrate. Neuronal cell number within the regions of interest was assessed using 3 10-µm sections stained with Cresyl fast violet (26).

### Neuropathological Image Analysis

For quantification of neuropathological lesions, the images were captured using a Zeiss Z1 microscope and MRc camera (Zeiss, Oberkochen, Germany) coupled to a PC with a motorized stage. The regions of interest were drawn at 1.25× magnification from the pial surface and using a line perpendicular to the white matter to encompass the immediate subjacent white matter, thereby covering all of the cortical gray matter within the region. Dissector boxes were placed in a uniform and unbiased way within the region of interest, with 10–15 frames captured at 10× magnification per region of interest for densitometric analysis. The images were analyzed using ImageJ 1.48v analysis software (Java 1.6.0_20 [64-bit]: http://imagej.nih.gov/ij, accessed May 29, 2019). The mean percentage area stained for each frame was determined using the red-green-blue (RGB) thresholds, which were optimized manually for each antibody to eliminate the detection of any nonspecific background staining, with thresholds set at a level that was reached by immunopositive pathological structures only. The mean percentage area stained per case was calculated from the mean values obtained across all images taken. For Cresyl fast violet-stained sections the same regions of interest were used as for quantification of neuropathological lesions, with the images captured using 63x oil immersion objective (26). Three 10-μm sections were used for each case to ensure adequate sampling. Differentiation of neuronal cells from glia was based on standard morphological criteria (27, 28). We used a systematic approach that employed an adaptation of stereology software to enable cell counts within a specified area, which was defined at 1.25× magnification, with 20–30 dissector frames randomly placed within the region of interest. The number of frames used was determined on the basis of the pilot study, generating sufficiently low coefficient of error. A systematic, randomly oriented point grid was superimposed over each image, with neurons counted within a dissector frame of known dimensions, generating an estimated neuronal number (mm^2^).

### SPECT Image Analysis

As previously described (24), each image volume was registered to standard MNI (Montreal Neurological Institute, http://www.bic.mni.mcgill.ca) space using statistical parametric mapping (SPM2, Wellcome Department of Imaging Neuroscience, London, UK, http://www.fil.ion.ucl.ac.uk/spm). Mean intensities representing relative perfusion within standard regions of interest were then calculated for each scan using the marsbar SPM toolbox (http://marsbar.sourceforge.net/) (29). Regions included the PHG, precuneus, cuneus, ACC, MCC, and PCC from the Desikan atlas (30) in freesurfer. These regions were smoothed by 8 mm FWHM Gaussian and thresholded to match the resolution of the SPECT. Intensities are presented relative to whole brain uptake. Data were taken from the right hemisphere only to match the postmortem data. The mean intensities for perfusion in the SPM2-defined regions of interest were used whereby the mean perfusion values for PCC were divided by the mean values in the Pr/Cu to derive the CIS ratio from the ^99m^Tc-HMPAO SPECT perfusion data similar to the approach used previously (10, 17).

### MRI Image Analysis

Volumetric estimates (mm^3^) of the cingulate substructures (i.e. ACC, MCC, and PCC) were then derived for each individual from data obtained from cortical reconstructions/segmentation of MRI scans using the FreeSurfer image analysis suite (v 5.1, http://surfer.nmr.mgh.harvard.edu/), the technical details of which have been described in prior reports (30–32).

### Statistical Analysis

Statistical analysis was undertaken in SPSS Statistics version 23.0. Distributional assumptions for outcome measures were assessed using the Shapiro–Wilk test. The nonparametric tests were applied for the neuropathological data analysis, as it did not meet the normal distribution criteria. The differences between multiple disease groups were assessed using Kruskal–Wallis test, and Friedman’s ANOVA for paired analysis. The effect sizes for group differences were assessed using Wilcoxon test. Correlation analyses were carried out using Spearman’s correlation coefficient *rho*. The relationship between CIS ratios, pathological, clinical and densitometric variables were assessed using linear regression analyses. Group comparisons within the regions of interest for cerebral perfusion, volumetric and densitometric data were carried out using analysis of variance (ANOVA), with reported p values adjusted for multiple comparisons using Bonferroni posthoc test.

## RESULTS

### Pathology

No significant difference was found in the gender (p =* *0.701), age at diagnosis (p =* *0.200), and postmortem delay (p =* *0.413) between the groups. A significant difference was observed in the age at death between groups, with DLB patients showing significantly younger age at death compared with controls (p =* *0.012) and AD (p =* *0.030; Supplementary DataTable S1).

**FIGURE 2.**
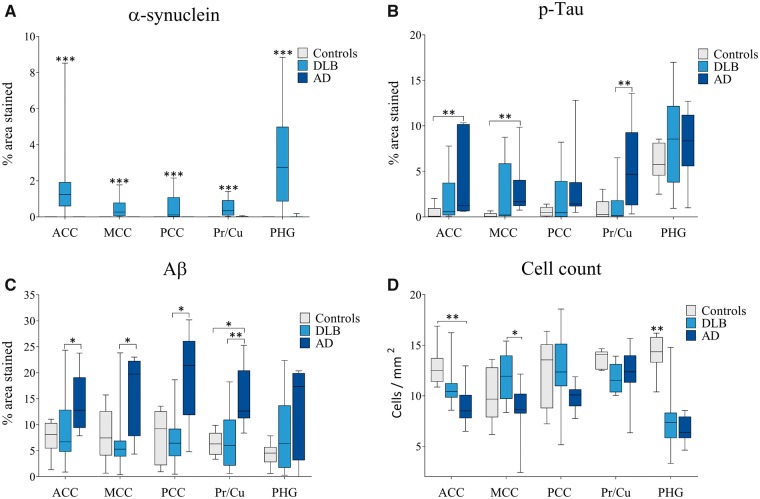
Pathological and cell number changes in cingulate subregions, precuneus/cuneus and parahippocampal gyrus in older normal, dementia with Lewy bodies (DLB), and Alzheimer disease (AD) donors. Postmortem brain tissue from DLB, AD, and control donors was sampled in specific brain regions associated with the cingulate island sign and 10-μm-thick tissue sections immunohistochemically stained to demonstrate neurodegenerative pathology, or stained using standard cresyl fast violet stain to demonstrate cellular morphology. Pathological burden (% area stained) was determined using computer-aided image analysis of **(A)** α-synuclein; **(B)** phosphorylated Tau (p-Tau); **(C)** amyloid-β (Aβ); and a stereological approach used to determine **(D)** neuronal number (mm^2^) in anterior cingulate cortex (ACC), midcingulate cortex (MCC), posterior cingulate cortex (PCC), precuneus/cuneus (Pr/Cu), and parahippocampal gyrus (PHG). Bars represent mean and SD (controls n = 6; DLB n = 12; and AD n = 9). α-Synuclein was significantly higher in DLB in all regions studied compared with both AD and control groups **(A)**. Phosphorylated tau burden was significantly higher in AD compared with controls in ACC and MCC **(B)**. Aβ burden was also higher in AD compared with DLB in all cingulate regions but did not differ from controls **(C)**, whereas cell count in AD ACC was reduced compared with control and in MCC compared with DLB (significance levels set at *p < 0.05, **p < 0.01, and ***p < 0.001). Box plots represent the median (midline) and quartile ranges, with error bars representing the minimum and maximum values.

Alpha-synuclein pathology was significantly higher in DLB patients compared with controls and AD in ACC, MCC, PCC, Pr/Cu, and PHG (p < 0.001; Fig. 2A). DLB patients showed significantly different α-synuclein burden distributions between cingulate subregions (*χ*^2^(2) = 6.000, p* *=* *0.050), with significantly higher α-synuclein burden observed in ACC compared with PCC (p* *=* *0.043). No significant differences were observed in α-synuclein burden between cingulate subregions in controls and AD (Supplementary DataFig. S1).

Phosphorylated Tau pathological burden was significantly different in ACC (*H*(2) = 8.766, p =* *0.012), MCC (*H*(2) = 8.635, p =* *0.013), and Pr/Cu (*H*(3) = 9.863, p =* *0.007) across disease groups. AD patients showed significantly higher p-Tau burden compared with DLB in Pr/Cu (p =* *0.010), and compared with controls in ACC and MCC (p =* *0.010; Fig. 2B). NFT Braak stage was significantly higher in AD patients compared with controls and DLB (p <* *0.001; Supplementary DataTable S1). No significant difference was observed in cingulate regional p-Tau pathology distribution within DLB, AD, or control groups (Supplementary DataFig. S2).

Amyloid-β pathological burden was significantly different in ACC, MCC, PCC, and Pr/Cu across disease groups. AD patients showed significantly higher Aβ burden compared with DLB in ACC (p =* *0.046), MCC (p =* *0.025), PCC (p =* *0.015), and Pr/Cu (p = 0.008), as well as compared with controls in Pr/Cu (p =* *0.017; Fig. 2C). DLB patients showed significantly different Aβ burden distribution between cingulate subregions (*χ*^2^(2) = 8.667, p =* *0.013), with significantly higher Aβ burden observed in ACC compared with MCC (p =* *0.013). No significant changes were observed in cingulate regional Aβ pathology distribution within AD or control groups (Supplementary DataFig. S3).

Neuronal density (neurons per mm^2^) was significantly higher in controls compared with AD in ACC (p =* *0.011) and PHG (p =* *0.003), and in controls compared with DLB in PHG (p =* *0.010). Significantly higher neuronal numbers were also observed in MCC in DLB compared with AD (p =* *0.025; Fig. 2D). In DLB group the cell density was significantly greater in PCC compared with ACC (p =* *0.043).

### Imaging

**FIGURE 3.**
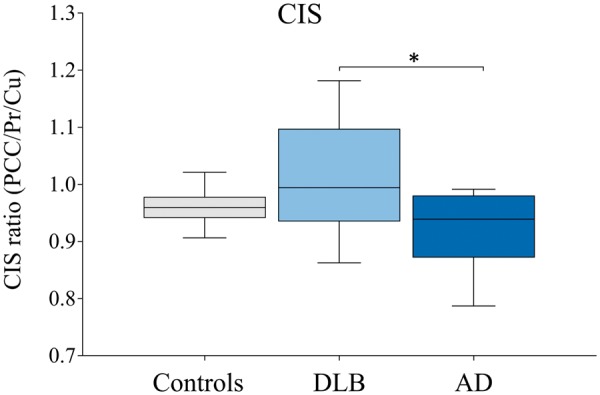
^99m^Tc-HMPAO SPECT derived cingulate island sign (CIS) ratio in older normal, dementia with Lewy bodies (DLB), and Alzheimer disease (AD) donors. ^99m^Tc-HMPAO SPECT imaging was performed in DLB (n = 12), AD (n = 9) patients or controls (n = 6) and images used to derive CIS values based on the ratio of posterior cingulate cortex (PCC) to precuneus/cuneus (Pr/Cu) perfusion values. Box plots of the CIS ratio represent the median (midline) and quartile ranges, with error bars representing the minimum and maximum values. DLB patients showed higher CIS ratio than AD patients (*p < 0.05), but were not significantly different than control older individuals.

No significant difference was observed in the disease duration at the time of SPECT (p =* *0.592) between DLB and AD. MMSE scores at the time of SPECT were significantly higher in controls compared with DLB (p =* *0.003) and AD (p =* *0.005), whereas UPDRS scores were significantly higher in DLB patients compared with controls (p =* *0.006) and AD (p =* *0.012). The interval between SPECT and death was significantly longer in controls (p =* *0.001) and AD patients (p =* *0.046) compared with DLB (Supplementary DataTable S1). ^99m^Tc-HMPAO SPECT uptake was not significantly different in any regions of interest between the groups (Table). Paired analysis within the DLB group showed significantly higher ^99m^Tc-HMPAO SPECT uptake in PCC (p =* *0.045) and MCC (p =* *0.006) compared with ACC. ^99m^Tc-HMPAO SPECT uptake in the AD group was also significantly higher in MCC (p =* *0.012), but not PCC (p =* *0.148) compared with ACC. No significant difference in ^99m^Tc-HMPAO SPECT uptake between cingulate subregions was observed in control patients. MRI volumes were significantly different in PCC between groups (*F*(2, 21)* *=* *6.172, p =* *0.008), with significantly higher PCC volume observed in controls compared with AD (p =* *0.009; Table). The CIS ratios were significantly higher in DLB patients compared with AD (p =* *0.041; Fig. 3).

**TABLE.** ^99m^Tc-HMPAO SPECT and MRI Imaging Data

	Controls	DLB	AD	Test Statistic
***HMPAO-SPECT***	(n = 6)	(n = 12)	(n = 9)	
ACC	72.3 (10.37)	69.9 (7.6)	69.5 (4.7)	*F*(2, 24) = 0.263, p = 0.771
MCC	76.8 (7.1)	75.4 (8.1)	75.3 (2.5)	*F*(2, 24) = 0.123, p = 0.885
PCC	76.2 (5.1)	76.2 (3.4)	72.4 (4.1)	*F*(2, 24) = 2.257, p = 0.126
Pr/Cu	78.2 (3.9)	73.9 (6.4)	78.7 (4.9)	*F*(2, 24) = 2.478, p = 0.105
PHG	70.3 (3.3)	71.9 (3.7)	67.4 (6.1)	*H*(2) = 4.117, p = 0.128
***MRI volume mm^3^***	(n = 6)	(n = 10)	(n = 8)	
ACC	1685.7 (829.3)	2170 (411.3)	1665.8 (522.6)	*F*(2, 21) = 0.673, p = 0.521
MCC	2092.2 (668.3)	1977.1 (648.2)	2158.6 (473)	*F*(2, 21) = 0.489, p = 0.620
PCC	2907.8 (779.7)	2673.1 (556.5)	1905.4 (368.4)	*F*(2, 21) = 6.172, p = 0.008; **(p** **=** 0.009)^a^**; (p = 0.059)^b^

^99m^Tc-HMPAO SPECT imaging or 3T structural MRI was performed in dementia with Lewy bodies (DLB), Alzheimer’s disease (AD) patients and controls. Values are expressed as the mean (± SD) for the anterior cingulate cortex (ACC), midcingulate cortex (MCC), posterior cingulate cortex (PCC), precuneus/cuneus (Pr/Cu) and parahippocampal gyrus (PHG). No region specific changes were seen between groups for ^99m^Tc-HMPAO SPECT although a significant difference was identified between AD and controls in PCC volume (Test Statistic: Bold p values indicate significant differences postBonferroni correction; [Control > AD]^a^; (DLB > AD)^b^.) Pairwise comparison of cingulate subregions in DLB showed lower ACC perfusion compared with MCC (**p < 0.01) and PCC (*p < 0.05), and in AD lower ACC perfusion compared with MCC (*p = 0.05), but no regional differences in controls.

### Linear Regression Analyses

No significant correlations were observed between α-synuclein, p-Tau or Aβ pathology in PCC and Pr/Cu with CIS ratios, or ^99m^Tc-HMPAO SPECT uptake in PCC or Pr/Cu within DLB group. Furthermore, no significant correlation was observed between CIS ratios, ^99m^Tc-HMPAO SPECT uptake in PCC, or Pr/Cu with NFT Braak stage in the DLB group. The neurodegenerative pathology in PCC also did not show significant correlations between cell density and volume in PCC in DLB. Additionally, we saw no correlations between MMSE scores at the time of SPECT with CIS ratios, or disease duration at the time of SPECT in DLB (Supplementary DataFig. S4).

## DISCUSSION

The CIS is a supportive biomarker showing high sensitivity and specificity in differentiating DLB from AD (2), and has been suggested to be indicative of the lower NFT Braak stage in patients with DLB using FDG-PET (10), although the underlying pathological basis of the CIS is unknown. In this study we observed significantly higher CIS ratios in DLB compared with AD using ^99m^Tc-HMPAO SPECT, in line with previous FDG-PET studies (9, 12). Our findings indicate that the currently considered classic pathological indicators of neurodegeneration did not correlate with the preserved metabolism of the PCC in DLB.

Unsurprisingly, DLB patients had higher α-synuclein burden in all cingulate regions compared with AD and controls. Within the DLB group a significantly higher α-synuclein burden was observed in ACC compared with PCC. Similar to previous studies (33–36), we also observed reduced metabolic activity in the ACC in DLB patients compared with MCC and PCC (Table), suggesting relatively preserved perfusion in PCC—the basis of the CIS. We, however, did not observe significant correlations between levels of α-synuclein pathology in PCC with CIS or ^99m^Tc-HMPAO SPECT uptake in PCC. This suggests that it is the relative levels of α-synuclein pathology between ACC and PCC that relate to the metabolic differences that give the CIS—high levels of α-synuclein in ACC leading to lower metabolism relative to lower levels of α-synuclein pathology in the PCC and preserved metabolism. The lack of a direct relationship between pathology may indicate that there is a threshold effect, once certain levels of pathology are reached, metabolism begins to decline and metabolic imaging changes can be observed. More direct markers of metabolism that are downstream consequences of pathological change will be required to observe these metabolic changes and allow the relationships between pathology and metabolism to be explored.

AD patients had significantly higher p-Tau burden compared with controls in ACC and MCC, and compared with DLB in Pr/Cu. While other studies have observed a relationship between lower NFT Braak stage and higher CIS ratios in clinically diagnosed DLB patients (10, 37), we found no such correlation. We also failed to observe a relationship between tau pathological burden in PCC with CIS and perfusion in PCC. The CIS is not specific to DLB and has been shown to be present in posterior cortical atrophy (PCA) (38). A high NFT Braak stage is typically observed in PCA (39), which may reflect differential AD pathology distribution (40) and potentially supports our finding of no association between NFT pathology and the CIS.

While it is unclear how the patterns of hypometabolism in dementia relate to underlying neuropathology, it has been suggested that Aβ deposition in posterior cortical regions may result in reduced metabolic activity in AD (41). Aβ burden was significantly higher in AD patients in all cingulate subregions and Pr/Cu compared with DLB. No correlations between Aβ burden in PCC and CIS ratio or ^99m^Tc-HMPAO SPECT uptake in PCC have been observed, which may suggest that Aβ pathology does not contribute directly to the reduced metabolic activity in the PCC.

While some studies have suggested that posterior cortical hypometabolism may be driven by cell loss, as observed in AD patients (37), we did not observe a relationship between CIS ratio and cell density. Occipital hypometabolism is also a common feature in DLB (11, 24). This region does not show significant atrophy (42), and typically does not show major pathological changes (43), which may suggest that factors other than pathological burden, cell loss or atrophy play a role in reduced metabolism in specific brain structures. Since the CIS is a metabolic marker, direct determination of underlying changes in metabolism in tissue may be more suitable. Markers such as mitochondrial enzymes or structural proteins are indicators of metabolic activity and may represent direct indicators of HMPAO retention. Similarly, synaptic activity is closely correlated with metabolism (44) and specific synaptic markers may provide suitable pathological markers of perfusion given the known loss of synapses in neurodegenerative disorders (45). Further work using such markers is therefore warranted to identify the basis of the CIS.

Hippocampal atrophy and PCC hypometabolism are common features of AD (23, 24). The hypometabolism of the PCC in AD and also lateral parietal cortex is thought to reflect disrupted inputs from the hippocampus (23, 46). While some studies have shown hippocampal atrophy to be associated with white matter changes in the cingulum in AD (47, 48), other studies have found that global rather than hippocampal atrophy correlated with white matter changes (23). The PCC is part of the default mode network and has high metabolic demands, which may suggest it has a higher vulnerability to damage. Whether the disruption of the cingulum precedes or is consequent upon hippocampal atrophy or PCC hypometabolism is not clear. A recent study has observed temporal changes in the CIS, with CIS in DLB being present at early disease stages and CIS diminishing with disease progression (49), however, we did not observe a relationship between CIS and MMSE scores, or disease duration at the time of SPECT. In the current study, we saw no significant changes in perfusion between groups in the regions of interest (Table). This is similar to previous work, which demonstrated significant changes in perfusion generally in frontal and temporal regions in AD, and in frontal and occipital regions in DLB compared with older control individuals (24). In DLB there was a trend toward reduction in perfusion (p =* *0.057) in the Pr/Cu in line with other studies (24) and similarly in AD a trend toward reduced perfusion in the PCC (p =* *0.158), although much larger groups sizes would be required to show this definitively. At an individual level the CIS, which depends on the ratio of PCC and Pr/Cu perfusion, allows the distinction to be made between the different disorders (Fig. 3).

The study has a number of strengths, including a clinically well-characterized cohort with neuropathological assessment and antemortem SPECT imaging similar in size to previously published studies. In contrast to other studies that used PET ligands for the visualization of tau in AD and DLB patients (10, 50), we were unable to identify a relationship between CIS and quantitative tau pathological burden. Using a combination of biopsy and follow up autopsy investigation, 1 study has demonstrated that plaque and neurofibrillary tangle density in AD patients changes very little between biopsy and death (51). This might suggest that other measures such as synaptic loss and altered metabolism are associated with patient decline, with neuropathology being an initiator of this cascade, which once activated is self-sustaining and relatively independent of amyloid and tau pathology. This may provide a reason why we did not identify a relationship between CIS and postmortem quantitative neuropathology. Additionally, while we detected CIS in our DLB patients using ^99m^Tc-HMPAO SPECT, FDG-PET imaging has been suggested to be more sensitive in detecting the CIS (12). Our use of ^99m^Tc-HMPAO SPECT may not have detected subtle changes in metabolism identifiable through FDG-PET. Furthermore, SPECT imaging in the current study was performed at different time points within the disease history, which might influence the results since previous studies have suggested that CIS is not present in advanced patients of DLB (49), although we saw no such correlation in this study. In summary, our results indicate that neurodegenerative pathology does not correlate with the CIS and preserved metabolism in the PCC in DLB. Further investigations are needed to determine whether metabolic deficits are attributable to mitochondrial changes, or a loss/dysfunction of synapses in the PCC and if these relate to the CIS.

## Supplementary Material

Supplement_Material_nlz047

## Notes

This project is supported by a studentship through the Alzheimer’s Society Doctoral Training Centre. Tissue for this study was provided by the Newcastle Brain Tissue Resource, which is funded in part by a grant from the UK Medical Research Council and the Brains for Dementia research, a joint venture between Alzheimer’s Society and Alzheimer’s Research UK. The research was also partly funded by the National Institute for Health Research (NIHR) Newcastle Biomedical Research Centre based at Newcastle Hospitals NHS Foundation Trust and Newcastle University.
