# Supplementary material for: Neuropathological Changes in Dementia With Lewy Bodies and the Cingulate Island Sign
Source: J Neuropathol Exp Neurol. 2019 May 24;78(8):717–24. doi: 10.1093/jnen/nlz047 (PMC6640897; doi:10.1093/jnen/nlz047)
Supplement: Supplement_Material_nlz047 [file supplement_material_nlz047.zip › Supplementary Table 1. Demographic data..docx]

**Supplementary Table 1. Demographic data.**

|  | **Controls**  (n=6) | **DLB**  (n=12) | **AD**  (n=9) | **Test statistic** |
| --- | --- | --- | --- | --- |
| **Gender (M:F)** | 4 : 2 | 7 : 5 | 4 : 5 | *F*(2, 24)=0.361, *p*=0.701 |
| **Age at diagnosis** | N/A | 73.25 (6.92) | 76.89 (5.62) | *t*=1.289, *p*=0.200 |
| **Age at death** | 90.16 (8.0) | 79.17 (6.82) | 87.67 (6.16) | *F*(2, 24)=6.602, *p*=0.005; **(*p*=0.012)ᵅ***; **(*p*=0.030)ᵇ*** |
| **Disease durarion (years)** | N/A | 6.16 (2.48) | 10.25 (3.85) | *t*=2.902, ***p*=0.022ᵇ*** |
| **Disease durarion at the time of SPECT** | N/A | 25.92 (23.3) | 30.7 (17.53) | *t*=0.546, *p*=0.592 |
| **MMSE at the time of SPECT** | 27.3 (25.76-28.91) | 18.42 (15.55-21.29) | 18.55 (15.1-22.1) | *F*(2, 24)=8.253, *p*=0.002; **(*p*=0.003)ᵅ****; **(*p*=0.005)ᶜ**** |
| **UPDRS at the time of SPECT** | 4 (1.68-6.32) | 21.08 (10.56-31.61) | 4.89 (3.09-6.69) | *H*(2)=13.048, *p=*0.001; **(*p*=0.006)ᵉ****; **(*p*=0.012)ᶠ*** |
| **Last MMSE** | 25.5 (23.14-27.86) | 9.25 (5.64-12.86) | 8.0 (2.24-13.75) | *H*(2)=12.836, *p=*0.002; **(*p*=0.008)ᵅ** (*p=*0.002)ᶜ**** |
| **Last UPDRS** | 9.5 (0.35-18.65) | 32.5 (24.14-40.85) | 12.88 (8.35-17.4) | *H*(2)=12.920, *p*=0.002; **(*p*=0.004)ᵉ**; (*p*=0.019)ᶠ*** |
| **Interval between SPECT to death** | 13 (10.14-15.86) | 4.17 (3.01-5.32) | 8.56 (6.15-10.95) | *H*(2)=15.323, *p<*0.001; **(*p*=0.001)ᵅ***; (*p*=0.046)ᵇ*** |
| **NFT Braak stage** | 2.3 (1.5-3.2) | 3.58 (2.7-4.4) | 5.7 (5.3-6.0) | *F* (2, 24)=16.279, *p*<0.001; **(*p=*0.001)ᵇ*****; **(*p<*0.001)ᵈ***** |
| **Post-mortem delay** | 66.83 (35.52) | 52.0 (30.64) | 45.33 (26.09) | *F* (2, 24)=0.918, *p*=0.413 |

**Supplementary Table 1. Demographic data**. Values expressed in mean (SD). Mini-Mental State Examination (MMSE), Unified Parkinson's Disease Rating Scale (UPDRS) and neurofibrillary tangle (NFT) Braak (mean and 95%CI). Bold *p*-values indicate significant differences post-Bonferroni correction. Dementia with Lewy bodies (DLB), Alzheimer’s disease (AD); (Control > DLB)ᵅ; (AD > DLB)ᵇ; (Control > AD)ᶜ; (AD > Control)ᵈ; (DLB>Controls)ᵉ; (DLB>AD)ᶠ. Disease durarion at the time of single-photon emission tomography (SPECT) and interval between SPECT to death (months).
