# Supplementary figures and images for: Neuropathological Changes in Dementia With Lewy Bodies and the Cingulate Island Sign
Source: J Neuropathol Exp Neurol. 2019 May 24;78(8):717–24. doi: 10.1093/jnen/nlz047 (PMC6640897; doi:10.1093/jnen/nlz047)

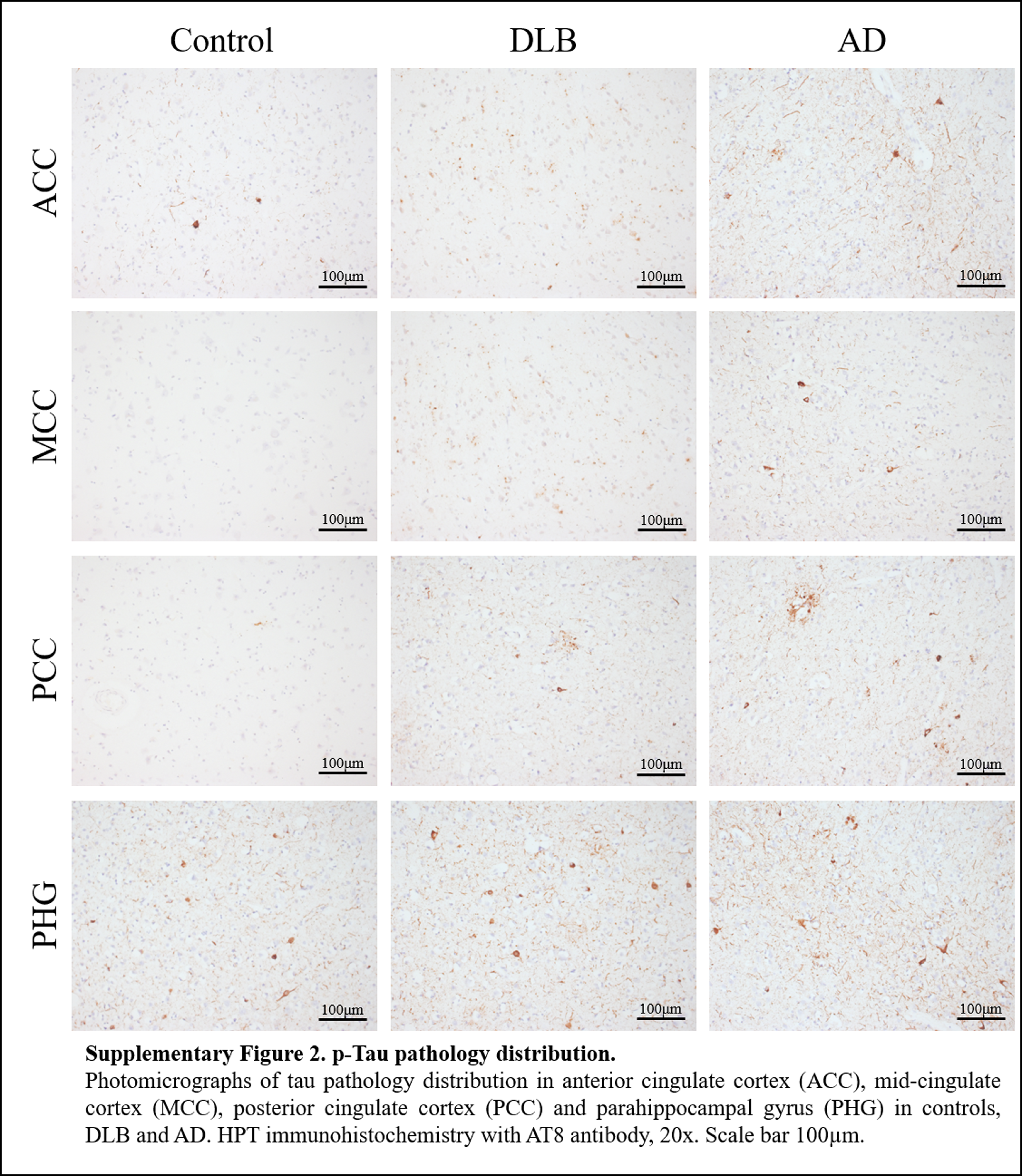

Supplement: Supplement_Material_nlz047 [file supplement_material_nlz047.zip › Supplementary Figure 2. p-Tau pathology.tif]

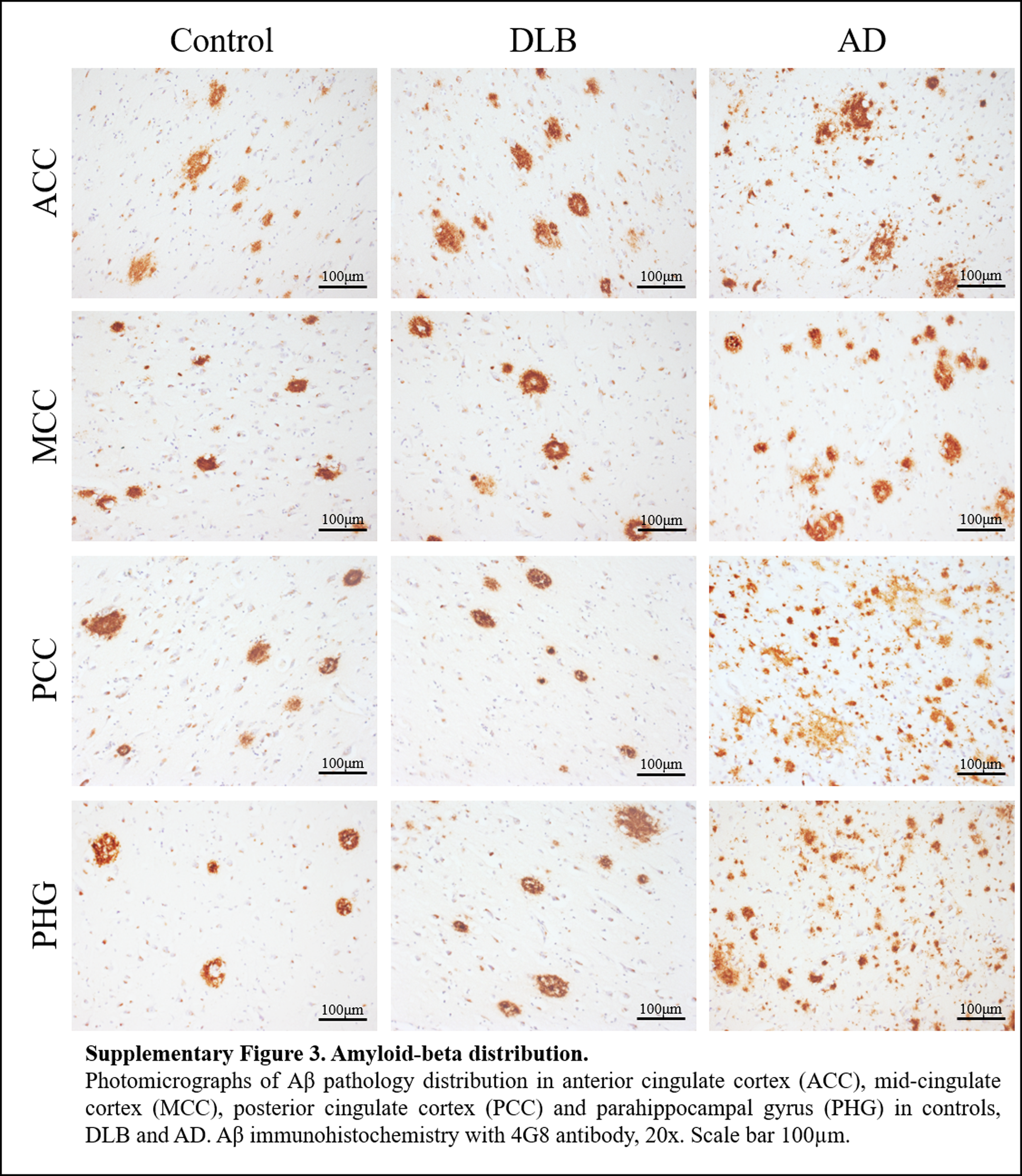

Supplement: Supplement_Material_nlz047 [file supplement_material_nlz047.zip › Supplementary Figure 3. Amyloid-beta.tif]

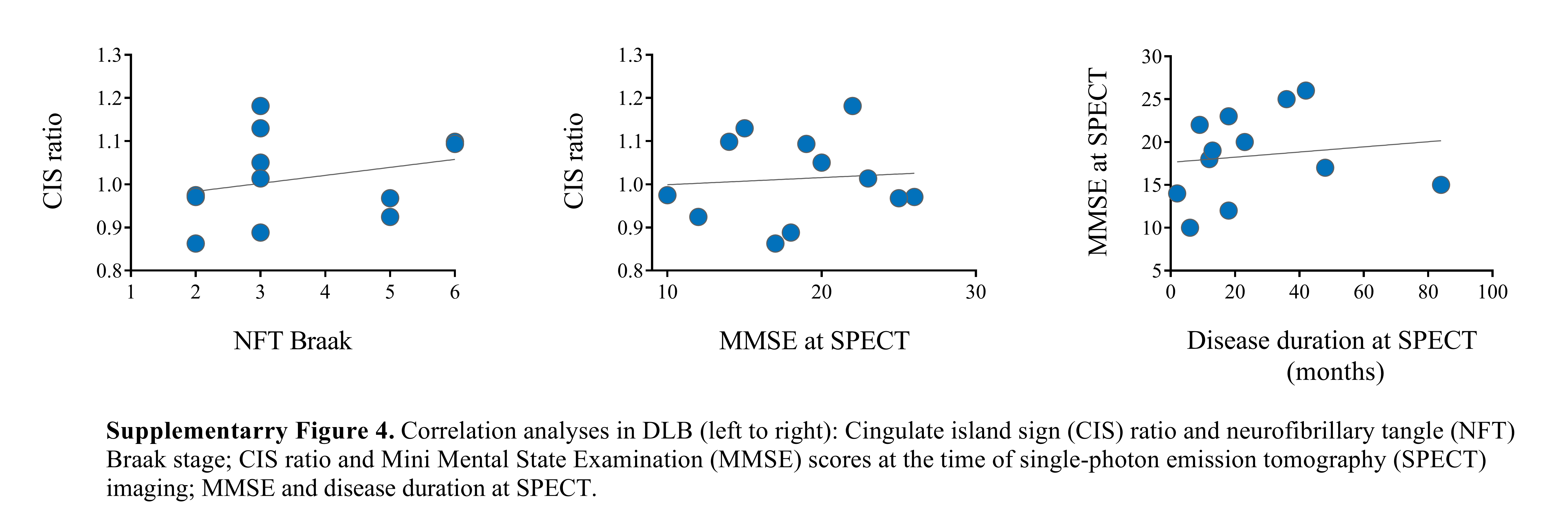

Supplement: Supplement_Material_nlz047 [file supplement_material_nlz047.zip › Supplementary Figure 4. Correlation analyses.tif]
